# Thermal fluctuations of haemoglobin from different species: adaptation to temperature via conformational dynamics

**DOI:** 10.1098/rsif.2012.0364

**Published:** 2012-06-13

**Authors:** A. M. Stadler, C. J. Garvey, A. Bocahut, S. Sacquin-Mora, I. Digel, G. J. Schneider, F. Natali, G. M. Artmann, G. Zaccai

**Affiliations:** 1 Institute for Complex Systems (ICS-5: Molecular Biophysics), Forschungszentrum Jülich, Jülich, Germany; 2 Jülich Center for Neutron Science (JCNS-1) and Institute for Complex Systems (ICS-1), Forschungszentrum Jülich, Jülich, Germany; 3 Australian Nuclear Science and Technology Organisation, Locked Bag 2001, Kirrawee DC, New South Wales 2232, Australia; 4 Laboratoire de Biochimie Théorique-CNRS UPR9080, Institut de Biologie Physico-Chimique, 13, rue Pierre et Marie Curie, 75005 Paris, France; 5 FH Aachen, Institute for Bioengineering, Heinrich-Mußmann-Straße 1, 52428 Jülich, Germany; 6 Jülich Centre for Neutron Science JCNS, Forschungszentrum Jülich, Outstation at FRM II, Lichtenbergstraße 1, 85747 Garching, Germany; 7 CNR-IOM, OGG c/o Institut Laue-Langevin, 6 rue Jules Horowitz, 38042 Grenoble, France; 8 Institut Laue-Langevin and CNRS, 6 rue Jules Horowitz, 38042 Grenoble, France

**Keywords:** haemoglobin, thermodynamic stability, protein dynamics, incoherent neutron scattering, coarse-grained Brownian dynamics simulations, circular dichroism

## Abstract

Thermodynamic stability, configurational motions and internal forces of haemoglobin (Hb) of three endotherms (platypus, *Ornithorhynchus anatinus*; domestic chicken, *Gallus gallus domesticus* and human, *Homo sapiens*) and an ectotherm (salt water crocodile, *Crocodylus porosus*) were investigated using circular dichroism, incoherent elastic neutron scattering and coarse-grained Brownian dynamics simulations. The experimental results from Hb solutions revealed a direct correlation between protein resilience, melting temperature and average body temperature of the different species on the 0.1 ns time scale. Molecular forces appeared to be adapted to permit conformational fluctuations with a root mean square displacement close to 1.2 Å at the corresponding average body temperature of the endotherms. Strong forces within crocodile Hb maintain the amplitudes of motion within a narrow limit over the entire temperature range in which the animal lives. In fully hydrated powder samples of human and chicken, Hb mean square displacements and effective force constants on the 1 ns time scale showed no differences over the whole temperature range from 10 to 300 K, in contrast to the solution case. A complementary result of the study, therefore, is that one hydration layer is not sufficient to activate all conformational fluctuations of Hb in the pico- to nanosecond time scale which might be relevant for biological function. Coarse-grained Brownian dynamics simulations permitted to explore residue-specific effects. They indicated that temperature sensing of human and chicken Hb occurs mainly at residues lining internal cavities in the β-subunits.

## 1. Introduction

Haemoglobin (Hb) is the major macromolecular component of red blood cells (RBCs). The protein forms a tetramer under physiological conditions of the two so-called α- and two β-chains. Each subunit acts as a scaffold for one haem-group, which can bind oxygen reversibly. One of the biological functions of RBCs is to transport oxygen from the lung to the tissues. Only higher vertebrates produce these highly specialized cells for oxygen transport in their bodies. Higher vertebrates can be subdivided into two sub-classes: endotherms—such as mammals or birds—which maintain a constant body temperature mainly through regulation of their internal metabolism, and ectotherms—such as reptiles or amphibians—whose body temperature is regulated by the environment.

The mechanical properties of single human RBCs have been studied by micropipette aspiration experiments as a function of temperature [1]. A passage transition occurring within a very narrow temperature range has been found in these experiments. Below a temperature of 36.4 ± 0.4°C, all aspirated RBCs blocked the pipette tip (diameter of the pipette approx. 1.3 µm) and were just compressed in volume. The Hb concentration within the RBCs, in this case, reached very high values up to approximately 0.5 g ml^−1^, whereas under physiological conditions, the cellular Hb concentration is approximately 0.33 g ml^−1^ [1]. Above the passage transition temperature, all aspirated cells entered the micropipette tip easily without any apparent resistance [1]. The passage transition temperature was surprisingly very close to human body temperature of approximately 37°C, which suggested a physiological relevance. Consecutively, the viscosity of highly concentrated Hb solutions from 0.33 to 0.5 g ml^−1^ was measured as a function of temperature [1]. A drop in the viscosity of the highly concentrated Hb solutions was found close to the passage transition temperature reminiscent of a colloidal gel-to-fluid transition. Hence, the passage transition of the RBCs was attributed to a more fluid Hb solution in the cells above the body temperature. Colloid osmotic pressure measurements on whole RBCs showed Hb aggregation starting at body temperature [2]. Small angle neutron scattering experiments revealed the presence of a large-scale superstructure with a mass fractal dimension of 2.67 in a concentrated Hb solution of 290 mg ml^−1^, which might provide the basis for body temperature-related Hb aggregation [3]. Further experiments have been performed to elucidate the molecular origin of the observed effects. Changes of secondary structure content of human Hb around the passage transition temperature were monitored by circular dichroism (CD), which revealed a significant loss of α-helical content of human Hb at 37.2 ± 0.6°C being again close both to body temperature and to the observed passage transition temperature [4]. The partial loss of secondary structure close to body temperature has been introduced as ‘partial unfolding’ in the literature. Furthermore, it has been investigated whether Hb molecules from endothermic vertebrates would show similar partial unfolding transitions close to their specific body temperatures [5]. Interestingly, this was indeed the case. The partial unfolding temperature of Hb from a large variety of species was directly correlated with the body temperature of the animal, ranging from 34°C for the duckbilled platypus, which was the animal with the lowest body temperature in that study, to 42°C for a bird, the spotted nutcracker, which had the highest body temperature in the study [5]. Incoherent quasi-elastic neutron scattering experiments on human RBCs and on concentrated human Hb solutions showed that the partially unfolded state is more flexible and softer than the folded state below body temperature [6,7]. In that sense, Hb appears to act as a molecular thermometer and to sense body temperature [2], which would be of outstanding interest and importance for biological, bioengineering and biomedical RBC research. As both the secondary structure content and the three-dimensional structure of Hb are highly similar [5], the mechanism underlying temperature sensing needs to be explored in the specific physico-chemical properties of the different amino acids and in their interactions [2]. One hypothesis would be that stronger internal forces in the structure of Hb would be responsible for the observed correlation between partial unfolding transitions and body temperatures. Thus, stronger internal forces might stabilize the structure of Hb and would be tuned very finely to respond to small differences in body temperature. However, to our knowledge, no studies are reported in the literature that investigated the relation between protein thermal stability, internal forces and conformational fluctuations in various Hb.

The aim of our study was to investigate whether internal dynamics and thermodynamic parameters of Hb from different species are adapted to body temperature, and to identify the thermo-sensitive amino acids on a residue-specific level. Neutron spectroscopy is a well-suited experimental technique to measure average fluctuations of proteins in the pico- to nanosecond time scale and in absolute units of a few ångströms [8–11]. Although significant differences were found in the internal dynamics of Hb in highly concentrated solution, the mean square displacements (MSD) of hydrated human and chicken Hb powder samples showed no differences over the whole temperature range from 10 to 300 K demonstrating that not all biologically relevant motions of Hb are activated with only one hydration layer. Coarse-grained Brownian dynamics simulations on the other hand are a complementary and very informative tool as they provide residue-resolved information on protein flexibility [12–14]. In order to obtain a detailed understanding of the experimental results and to explore residue-specific effects, coarse-grained Brownian dynamics simulations were performed on human and chicken Hb.

## 2. Material and methods

### 2.1. Sample preparation

Human Hb was purchased from Sigma-Aldrich. Blood samples of platypus, chicken and salt water crocodile were obtained from live animals. RBCs were harvested by centrifugation and washed several times with isotonic buffer. The platypus RBCs were then lysed by adding distilled water and centrifuged at 20 000 relative centrifugal force (rcf). Crocodile and chicken RBCs are nucleated and were lysed with a lysis buffer (0.5% Tris-HCl at pH 7.5) to avoid breaking the nuclear membrane. Nuclei were separated by centrifugation at 2800 rcf and the supernatant was spun at 20 000 rcf. The Hb solutions were dialysed against distilled water before lyophilization. For the neutron scattering experiments, the Hb powders were dissolved in 99.9 per cent D_2_O to exchange the labile H atoms and freeze dried afterwards.

For the experiments on the IN13 neutron spectrometer (see §2.2), highly concentrated Hb solutions were prepared. The dry Hb powders were placed in flat aluminium sample holders of beam path 0.20 or 0.30 mm. D_2_O buffer (0.1 M KCl, 61.3 mM K_2_HPO_4_, 5.33 mM KH_2_PO_4_, pD 7.4) was then added to a level of 1.1 g D_2_O/1 g Hb. The following amounts of Hb were used for the experiments on IN13: 23 and 75 mg platypus Hb, 118 mg crocodile Hb, 250 mg chicken and human Hb. Hydrated Hb powder samples were prepared for the experiments on the SPHERES neutron spectrometer (see §2.2). Human and chicken Hb were placed in flat aluminium sample holders and dried in a desiccator over silica gel until no further loss of weight was observed. The dried Hb powders were rehydrated to a level of 0.4 g D_2_O/1 g Hb in a saturated D_2_O atmosphere. The hydration level was determined by weighing the samples. Around 300 mg of human and chicken Hb were used for the experiments on SPHERES. The beam path of the sample holders was 0.30 mm.

Hb samples for CD spectroscopy were prepared at a concentration of 0.3 mg ml^−1^ in 20 mM K_2_HPO_4_/KH_2_PO_4_ buffer at pH 7.0.

### 2.2. Neutron scattering experiments

Neutron scattering was measured on the thermal neutron backscattering spectrometer IN13 at the Institut Laue-Langevin in Grenoble, France [15], and on the cold neutron backscattering spectrometer SPHERES at the FRM II in Garching, Germany [16]. The relation between the modulus of the scattering vector *q*, the incident neutron wavelength **λ** and the scattering angle 2**θ** is *q* = 4*π*/**λ** sin(**θ**) for neutrons without energy transfer. IN13 is characterized by an energy resolution of *ΔE* = 8 µeV (FWHM), and a scattering vector range of 0.2 < *q* < 4.9 Å^−1^. SPHERES is characterized by a very high-energy resolution of *ΔE* = 0.66 µeV (FWHM) and a scattering vector range of 0.17 < *q* < 1.84 Å^−1^. IN13 is sensitive to macromolecular motions in the space–time window of a few ångströms in approximately 0.1 ns, whereas SPHERES detects slower fluctuations with amplitudes of a few ångströms in approximately a few nanoseconds.

The neutron detectors of IN13 were calibrated with a vanadium measurement at 280 K. On SPHERES, the Hb powder samples were cooled to 10 K, and the low-temperature measurements were used for calibration. The incoherent contribution of D_2_O in the concentrated Hb solution samples was estimated to be less than 4 per cent of the total incoherent scattering [7] and is negligible for the hydrated powder samples. On IN13, the temperature was varied stepwise, while on SPHERES, the temperature was ramped up continuously from 10 to 300 K within 1 day, and measured data were binned into temperature steps of 5 K. Measured data of the empty sample holder were subtracted from the samples. No multiple scattering corrections were performed as the transmissions of all samples were above 0.9.

Mean square displacements 〈*u*^2^〉 were calculated within the Gaussian approximation according to
2.1
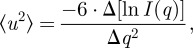

where *I*(*q*) represents the measured elastic intensity [9]. The 〈*u*^2^〉 were determined from the initial slope of the measured elastic intensity in the *q^2^*-range of 1.6 ≤ *q^2^* ≤ 3.5 Å^−2^ for IN13, and 0.36 ≤ *q^2^* ≤ 3.10 Å^−2^ for SPHERES, where the initial slope of the curves was found to be linear. The Gaussian approximation of protein dynamics [17] is mathematically identical to the Guinier approximation of small-angle scattering [18,19]. The Gaussian approximation in the strictest sense holds up to 〈*u*^2^〉 · *q*^2^∼2 for confined motions of all shapes [9]. However, protein dynamics is intrinsically anisotropic with motions of ellipsoidal shape and axial rations of 1 : 1 : 2 [20]. The Guinier approximation holds to larger values for such ellipsoidal shapes [19]. For protein dynamics, the Gaussian approximation was found to be valid for 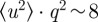 [21,22]. The 〈*u*^2^〉 values measured with IN13 in our study are in the range up to 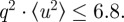 For SPHERES, they are below 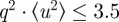.

Effective mean force constants 〈*k′*〉 that describe the mean resilience of the proteins were obtained from the slope of 〈*u*^2^〉 versus temperature *T* according to
2.2
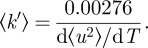

The units are chosen such that 〈*k′*〉 is in N m^−1^ when 〈*u*^2^〉 is in Å^2^ and *T* is in K [9,23].

### 2.3. Circular dichroism experiments and thermodynamic analysis

A J-810 spectropolarimeter (JASCO Corp., Tokyo, Japan) equipped with a temperature-controlled cuvette holder was used to record the CD spectra . The temperature-dependent loss of α-helical structure was determined from the CD signal at a constant wavelength of 222 nm. The samples were measured in 1 mm optical path quartz cuvettes under constant N_2_ flow at a heating rate of 1°C min^−1^. The raw data were corrected for pre- and post-transitional slopes. A van't Hoff analysis was used to determine the thermodynamic values of *ΔH* = *H*_unfolded_ − *H*_folded_ and *ΔS* = *S*_unfolded_ − *S*_folded_ between the unfolded and folded state at the melting temperature *T*_m_. The model assumes a two-state equilibrium between the unfolded and the folded state. In the unfolding transition region, the difference in free-energy *ΔG* was calculated using 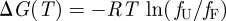 with the folded fraction *f*_F_, the unfolded fraction *f*_U_ = 1 − *f*_F_ and the molar gas constant *R.* The enthalpy and entropy differences at *T*_m_ were determined according to 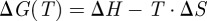 in the linear regime.

### 2.4. Sequence alignments

Protein amino acid sequences were obtained from the NCBI gene and protein databases (http://www.ncbi.nlm.nih.gov/). Multiple sequence alignments were performed using the Clustal W software [24]. The α-helical content of the subunits was calculated using the Jpred3 secondary structure prediction server [25]. Entry names and accession numbers of the sequences of the alpha- and beta-units are: human Hb, HBA_HUMAN P69905 and HBB_HUMAN P68871; platypus Hb, HBA_ORNAN P01979 and HBB_ORNAN P02111; chicken Hb, HBA_CHICK P01994 and HBB_CHICK P02112; crocodile Hb, HBA_CRONI P01998 and HBB_CRONI P02129.

### 2.5. Computer simulations

Coarse-grained Brownian dynamics simulations were carried out on the biological assemblies of the human and chicken Hb deoxy crystal structures (pdb access codes 2DN2 and 1HBR) using the ProPHet (probing protein heterogeneity) program [13,26,27]. The simulations used a coarse-grained protein model, in which each amino acid is represented by one pseudoatom located at the C_α_ position, and either one or two (for larger residues) pseudoatoms replacing the side chain (with the exception of Gly) [28]. Interactions between the pseudoatoms were treated according to the standard elastic network model [29]. The elastic network model is a simplification of the heterogeneity of internal protein forces as all pseudoatoms lying closer than 9 Å are joined with quadratic springs having the same force constant of 0.6 kcal mol^−1^ Å^−2^ = 0.42 N m^−1^. Springs are assumed to be relaxed in the crystallographic structure of the protein. Following earlier studies, which showed that the haem group had little influence on calculated force constants [13,27], we chose not to include the prosthetic group in the protein representation. The simulations used an implicit solvent representation via the diffusion and random displacement terms in the equation of motion [30], and hydrodynamic interactions were included through the diffusion tensor [31]. Further details regarding the simulation procedure can be found in Sacquin-Mora & Lavery [13] and Sacquin-Mora *et al*. [27].

The Brownian dynamics simulations were performed with 200 000 steps at an interval of 10 fs and at a temperature of 300 K. Effective force constants for displacing each particle *i* were calculated as
2.3
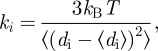

where the brackets indicate the average taken over the whole simulation, *k*_B_ is the Boltzmann constant and *d_i_* is the average distance of particle *i* from the other particles *j* in the protein, excluding the pseudoatoms, which belong to the same residue *m* to which also particle *i* belongs. The distances between the C_α_ pseudoatom of residue *m* and the C_α_ pseudoatoms of the adjacent residues *m* + 1 and *m* − 1 are not included in the average. The force constant associated with each residue *m* is taken to be the average of the force constants calculated according to equation (2.3) for each of the pseudoatoms *i* forming this residue. Within this framework, the mechanical properties of the protein are described at the residue level by its ‘rigidity profile’, that is, by the ordered sequence of the force constants calculated for each residue.

## 3. Results and discussion

Mechanisms of thermo-adaptation have been studied mainly for proteins from bacteria and archaea, which live at extremes of temperatures and are specially adapted to their optimal living conditions. Adaptation to high temperature can be achieved in various ways, for example, by stronger hydrogen bonding, clusters of charged amino acids or reduced internal cavities [32]. Hb on the other hand is a protein from higher vertebrates and it is not necessarily the case that mechanisms found in extremophiles determine thermosensation in Hb.

### 3.1. Multiple sequence alignments and secondary structure prediction

**Figure 1.**
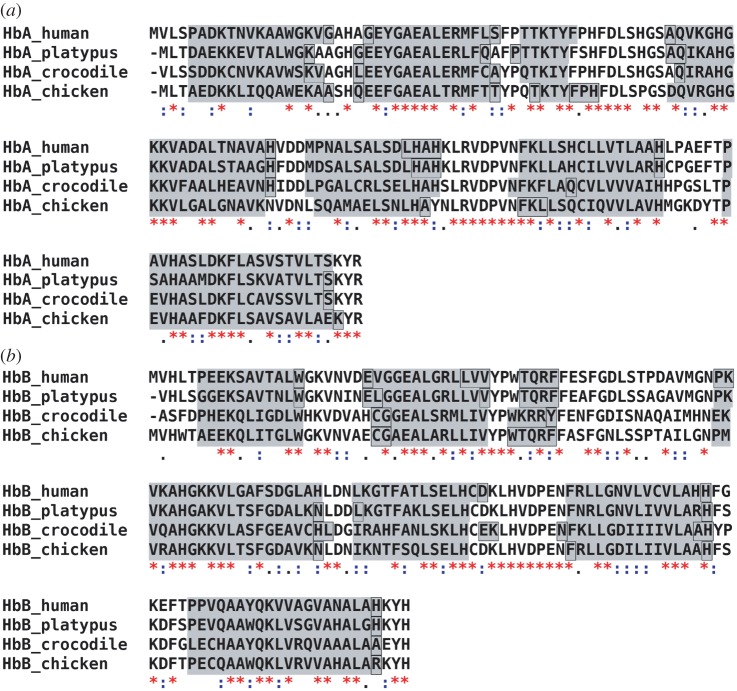
Amino acid sequence alignment of the (*a*) α- and (*b*) β-subunits of human, platypus, crocodile and chicken Hb. The amino acid similarity between all proteins is given as follows: asterisks indicate identical amino acids, colons indicate similar amino acids and empty space represents absence or low degree of similarity. The grey background indicates predicted α-helical contents of the subunits. Black frames label the parts of predicted helices where the prediction confidence was less than 50%. (Online version in colour.)

The structures of human and chicken Hb have been solved by X-ray crystallography [33,34]. Both proteins are highly similar in their structures. The crystal structures of salt water crocodile and platypus Hb have not yet been determined. We performed multiple sequence alignments using the Clustal W software [24] and predicted the α-helical regions from the aligned amino acid sequences using the JPred3 secondary structure prediction server [25]. The amino acid sequence of salt water crocodile is not published. As a compromise, we used the amino acid sequence of nile crocodile, which is a very close relative [35]. Both crocodile proteins are expected to be similar in their sequences and in their secondary structures. The aligned sequences and the predicted α-helical regions of all proteins are shown in figure 1. Seven α-helical regions are predicted both for the α- and β-subunits of all Hb proteins. Note that the crystal structures show eight helices. The length and location of the predicted regions are nearly identical for all proteins. The sequence alignments demonstrate that all proteins share a large amount of similar and identical amino acids. However, there are also a considerable number of non-similar amino acids distributed in the sequences. Regions with a larger number of non-similar amino acids are located before and in the first predicted helix of the α- and β-subunits, in the small loop between the predicted helices F and G of the α-subunit and in the large loop between the predicted helices C and D of the β-subunit. Although, a high similarity of the secondary structure of all proteins was found, we cannot infer from the sequence alignments which residues are responsible for differences occurring in the thermodynamic properties and in the conformational fluctuations. Protein thermal motions and thermodynamics depend on the local heterogeneous chemical environment and also on the interactions of different amino acids which might be separated in the sequence, but which are actually in close contact in the structure.

### 3.2. Thermodynamic analysis

**Figure 2.**
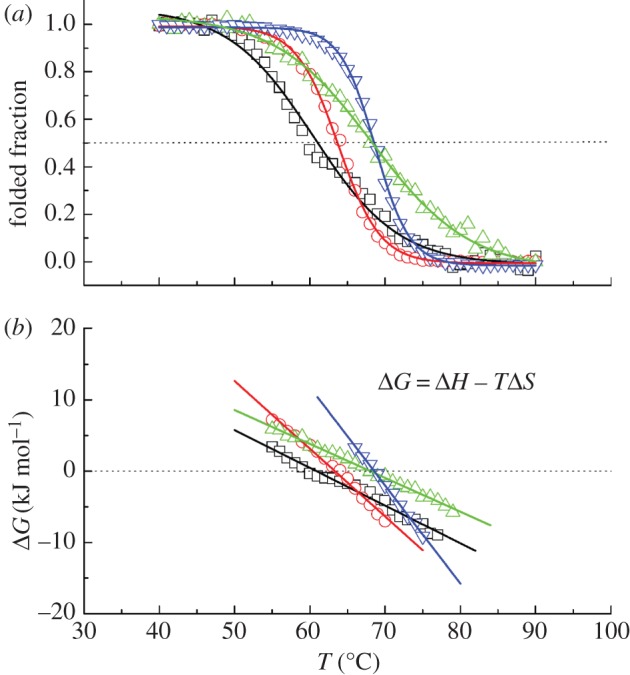
(*a*) Unfolding transitions of the investigated Hb samples. (*b*) Difference in free-energy *ΔG* between the unfolded and the folded state of the proteins in the transition region. Squares, platypus Hb; circles, human Hb; triangles, crocodile Hb; inverted triangles, chicken Hb. (Online version in colour.)

The thermal stability of Hb was studied by measuring the unfolding transitions using CD. We assumed a direct proportionality between the folded fraction of the protein and the recorded ellipticities. The obtained unfolding curves are shown in figure 2*a*, and the derived free-energy difference *ΔG* = *G*_unfolded_ − *G*_folded_ between the unfolded and the folded states in the transition region is given in figure 2*b*. The enthalpy and entropy differences *ΔH* and *ΔS* at the melting temperatures *T*_m_ were determined from *ΔG*. The thermodynamic parameters *T*_m_, *ΔH* and *ΔS* are summarized in table 1. The *T*_m_ of chicken and human Hb are in good agreement with melting temperatures of avian Hb (goose 68.2°C, chicken 67.6°C and turkey 67.8°C) and human Hb (64°C) as reported by Ajloo *et al*. [36]. To our knowledge, no melting temperatures of Hb of salt water crocodile or platypus are reported in the literature. Zerlin *et al*. [5] reported average body temperatures *T*_b_ of healthy platypus, human and chicken of 33.0 ± 1°C, 36.6 ± 0.2°C and 41.0 ± 0.5°C, respectively. For comparison, the body temperatures are also given in table 1. The errors of the body temperatures refer to statistical variations during repeated measurements of healthy animals under the same conditions. Obviously, the body temperature of an animal depends on its physical activity, on the place of the body where the measurement was made and even varies in the circadian cycle. For example, the body temperature of freely living platypuses was found to vary within ±3°C around the average value [37]. Body temperature can also vary between different animals of the same species. A statistical analysis of literature data was reported 34.4–37.8°C as a typical body temperature range of humans [38]. From a cautious point of view, the body temperature range of the different species should be considered more as ±3°C around the average value. The measured melting temperatures *T*_m_ of the three endotherms show a direct correlation with the reported average body temperatures (see figure 4). A similar correlation between body and environmental living temperatures was reported in the literature for eye lens crystallins of different vertebrates, ranging from an average living temperature of −1.9°C and a melting temperature of *T*_m_ = 47°C of the Antarctic teleost fish to an average living temperature of 47.0°C and a melting temperature of *T*_m_ = 67.4°C of the desert iguana lizard [39]. Crocodiles are in that sense a special case. As ectotherms, they do not metabolically maintain a constant body temperature. Instead, they regulate their body temperature by moving between water and land. In this way, they can keep their body temperature in a relatively narrow range by their behaviour. Grigg *et al*. [40] reported a body temperature range of salt water crocodiles between 25°C and 34°C. The melting temperature of crocodile Hb was found to be rather high, within the error bars identical to chicken Hb, although the physiological body temperature range is situated at comparatively low temperatures.

**Table 1.** Average body temperatures *T*_b_, thermodynamic parameters *T*_m_, *ΔH* and *ΔS* of the different Hb samples. The enthalpy and entropy differences between the unfolded and the folded states were determined at *T*_m_. The errors of *T*_m_ are around 0.5%, the errors of *ΔH* and *ΔS* are around 3%. The statistical errors of *T*_b_ of the endotherms are below 3%, body temperature ranges of up to ±3°C are reported.

	platypus Hb	human Hb	chicken Hb	crocodile Hb
*T*_b_ (°C)	33	36.6	41	25–34
*T*_m_ (°C)	60.5	63.8	68.7	68.3
*ΔH* (kJ mol^−1^) at *T*_m_	177	320	471	162
*ΔS* (kJ mol^−1^ K^−1^) at *T*_m_	0.529	0.950	1.379	0.476

Further information on the mechanisms of thermal stabilization can be obtained from the thermodynamic parameters *ΔH* and *ΔS* at *T*_m_. Concerning Hb from the endotherms, higher thermal stability is correlated with an increase of both *ΔH* and *ΔS* at *T*_m_. A larger value of *ΔH*(*T*_m_) shifts the free-energy difference *ΔG* to larger values and increases thermal stability. An increase of *ΔS*(*T*_m_) at the same time results in a steeper slope of *ΔG*, which results in a reduction of the melting temperature (figure 2*b*). As a consequence, the enthalpic component seems to dominate the thermodynamic stabilization of Hb from the endotherms. A different mechanism of thermal stabilization can be identified for crocodile Hb: Crocodile Hb has a high-melting temperature similar to that of chicken Hb, but the enthalpy and entropy differences at the melting temperature of crocodile Hb are nearly three times smaller than those of chicken Hb (compare table 1). Intriguingly, the values of *ΔH*(*T*_m_) and *ΔS*(*T*_m_) of crocodile Hb are even smaller than those of platypus Hb, which has the smallest melting temperature of the investigated proteins. The small value of *ΔH*(*T*_m_) of crocodile Hb destabilizes the folded state of the protein. The enthalpic destabilization is compensated by a small *ΔS*(*T*_m_) value, which stabilizes the folded state. The smaller entropy difference *ΔS* at *T*_m_ of crocodile Hb compared with that of platypus Hb results in a flatter slope of *ΔG* in the transition region, which shifts the *T*_m_ of crocodile Hb to a higher value. Indeed, the combination of enthalpic and entropic terms to protein stabilization is subtle and carefully counterbalanced for crocodile Hb to reach a high-melting temperature.

### 3.3. Incoherent elastic neutron scattering

**Figure 3.**
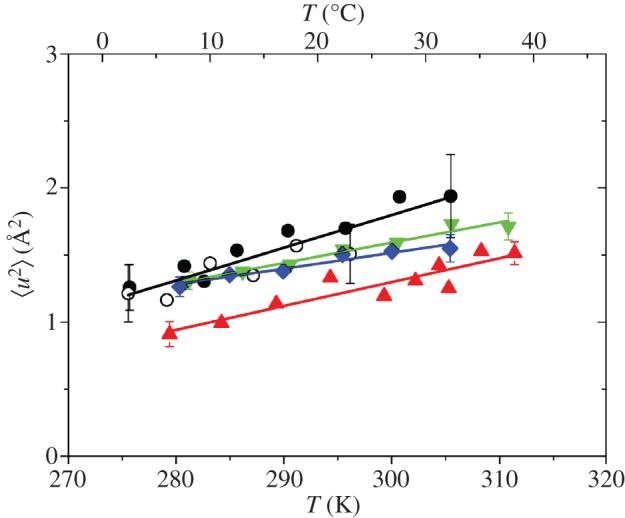
Mean square displacements 〈*u*^2^〉 of the highly concentrated Hb solutions as a function of temperature. Black circles, platypus Hb; red triangles, human Hb; green inverted triangles, crocodile Hb; blue diamonds, chicken Hb.

The Hb samples as highly concentrated solutions (1.1 g D_2_O/1 g dry Hb) were measured using the thermal neutron backscattering spectrometer IN13. The instrument is sensitive to macromolecular conformational motions on the time scale of a few 0.1 ns and with amplitudes of a few ångströms. The average MSD, 〈*u*^2^〉, were determined within the Gaussian approximation from the recorded intensities. Representative measured data of all samples are given in electronic supplementary material, figure S1. The determined MSD of all samples are shown in figure 3. Only a comparatively small amount of platypus Hb was available for the experiments, which explains the large errors. The measurement of platypus Hb was repeated with a sample prepared from a different animal as a control. The MSD of both samples overlap within the error bars, and the 〈*u*^2^〉 were combined for the analysis. Effective force constants 〈*k′*〉 and the MSD at the reported body temperatures [5] were determined from linear fits to the 〈*u*^2^〉. The obtained values of 〈*k′*〉 and the values of the root mean square displacements (RMSD), 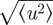, at the body temperatures of the three endotherms are reported in table 2.

**Table 2.** Force constants and root mean square displacements obtained from neutron scattering. Effective force constants 〈*k′*〉 and RMSDs at *T*_b_ of the concentrated Hb solution samples (IN13 data), and effective force constants 〈*k*_1_〉, 〈*k*_2_′〉, 〈*k*_3_′〉 of the hydrated powder samples (SPHERES data). The values of 〈*k*_1_〉, 〈*k*_2_′〉 and 〈*k*_3_′〉 were calculated in the temperature regions 10–100 K, 100–200 K and 252–292 K, respectively.

	platypus Hb	human Hb	chicken Hb	crocodile Hb
IN13				
〈*k′*〉 (N m^−1^)	0.11 ± 0.01	0.15 ± 0.02	0.23 ± 0.03	0.18 ± 0.02
RMSD at *T*_b_ (Å)	1.39 ± 0.11	1.22 ± 0.03	1.23 ± 0.04	—
SPHERES				
〈*k*_1_〉 (N m^−1^)	—	6.8 ± 1.2	5.6 ± 0.8	—
〈*k*_2_′〉 (N m^−1^)	—	1.0 ± 0.1	1.1 ± 0.1	—
〈*k*_3_′〉 (N m^−1^)	—	0.21 ± 0.02	0.24 ± 0.02	—

Tehei *et al*. [21] studied average macromolecular dynamics in psychrophilic, mesophilic, thermophilic and hyperthermophilic bacteria using elastic incoherent neutron scattering on IN13. The optimal growth temperatures of the different bacteria are 4°C for the psychrophile, 37°C for the mesophiles, 75°C for the thermophile and 85°C for the hyperthermophile [21]. Interestingly, the average macromolecular flexibility was found to be very similar at the optimal growth temperatures of the bacteria with a 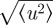 value close to 1.2 Å in support of an equivalent state hypothesis: i.e. a selection by evolution of similar average dynamics for the protein at physiological condition [21]. The resilience or ‘stiffness’ of the macromolecules increased from the psychrophilic to the hyperthermophilic macromolecules. The obtained effective force constants ranged from 〈*k′*〉 = 0.22 ± 0.03 N m^−1^ for the psychrophilic *Aquaspirillum arcticum*, to 0.43 ± 0.01 N m^−1^ and 0.40 ± 0.01 N m^−1^ for the mesophilic *Escherichia coli* and *Proteus mirabilis* and to 0.68 ± 0.11 N m^−1^ and 0.61 ± 0.01 N m^−1^ for the thermophilic *Thermus thermophilus* and hyperthermophilic *Aquifex pyrofilus* cells [21]. In a following study by the same authors on rabbit lactate dehydrogenase (LDH) and a hyperthermophilic archeal homologue, a 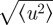 value of around 1.5 Å was reported at the corresponding temperatures of optimal catalytic activity. The rabbit LDH and the hyperthermophilic archeal homologue were characterized by a soft force constant of 〈*k′*〉 = 0.15 N m^−1^ and a very stiff force constant of 1.5 N m^−1^, respectively [22].

**Figure 4.**
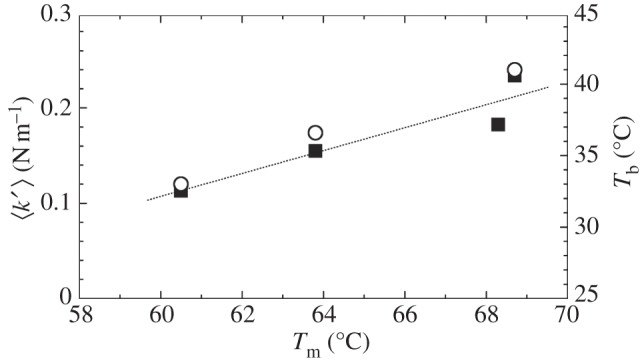
Melting temperatures *T*_m_, effective force constants 〈*k′*〉 and body temperatures *T*_b_ of the endotherms. The dashed line indicates a direct correlation between the results. Filled squares, effective force constants; open circles, body temperature.

We observed similar equivalent state behaviour for vertebrate Hb in our study with an adaption to physiological temperature. Platypus Hb has the softest structure, human Hb is stiffer and chicken Hb was found to be the stiffest protein. Crocodile Hb was found to be comparatively stiff, with a force constant between human and chicken Hb (compare table 2). The resilience of the Hb proteins is closer to that of rabbit LDH than to the resilience of the archeal or the bacterial proteins [21,22]. Stronger internal forces are indeed correlated with higher melting temperatures of the Hb of all species, and with the average body temperatures of Hb of the endotherms, shown in figure 4. Platypus Hb, which has the smallest effective force constant, shows the larges MSD of all measured proteins. However, both crocodile and chicken Hb, which are the stiffest proteins in this study, have 〈*u*^2^〉 values that are above those of the softer human Hb. The observations confirm that resilience and MSD are independent parameters related to the particular shape of the force field and that a general assumption, for example, that a stiffer protein is always less flexible than a softer protein is not valid.

We now compare the measured RMSD of the Hb molecules at the reported body temperatures *T*_b_ of the animals (table 2). The RMSD of human and chicken Hb at the respective body temperatures are very close to 1.2 Å. The RMSD of platypus Hb at *T*_b_ is slightly larger, 1.4 Å with a large error. These results suggest that a RMSD value close to 1.2 Å at *T*_b_ seems to be the optimal flexibility of the proteins at that temperature. RMSD of crocodile Hb varies between 1.2 Å at 25°C and 1.3 Å at 34°C, which is remarkably close to the average flexibility of 1.2 Å at the body temperatures of the other animals. Strong forces within crocodile Hb might keep the amplitudes of motion close to the optimal flexibility of the protein over the whole body temperature range of the animal. The obtained RMSD of Hb at body temperature is nearly identical to the RMSD of the macromolecules in the bacteria [21], but slightly smaller than the RMSD of the rabbit LDH and the hyperthermophilic archeal homologue [22].

**Figure 5.**
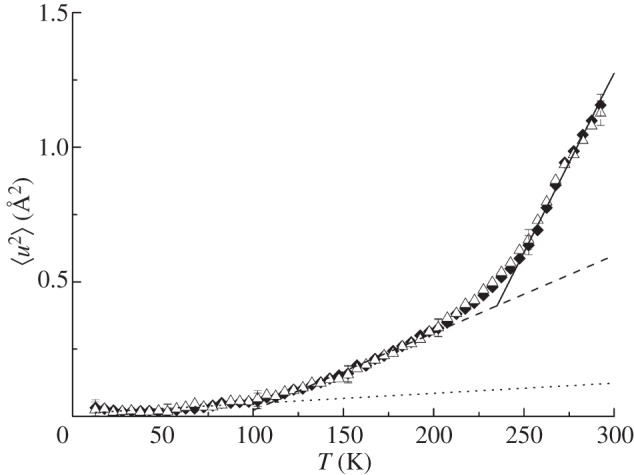
Mean square displacements 〈*u*^2^〉 of the hydrated Hb powder samples as a function of temperature. The dotted, dashed and solid lines are linear fits to the data in the temperature range from 10 to 100 K, from 100 to 200 K and from 252 to 292 K, respectively. The straight lines serve to guide the eye. Triangles, human Hb; diamonds, chicken Hb.

To investigate the role of hydration, we studied protein fluctuations of human and chicken Hb at different hydration levels. Hydrated protein powders at a hydration level of 0.4 g D_2_O/1 g dry protein corresponding to around one hydration layer are traditionally used to study protein dynamics using incoherent neutron scattering [41,42]. One hydration layer is assumed to be sufficient to activate anharmonic motions above around 180–240 K, while global protein diffusion is absent, which facilitates the interpretation of the measured MSD. The powder samples were measured using the cold neutron spectrometer SPHERES. The instrument has a high-energy resolution corresponding to observable motions in a space–time window of a few ångströms in a few nanoseconds. Representative measured data of both samples are given in electronic supplementary material, figure S2. The determined MSD of human and chicken Hb are shown in figure 5. Three linear regions are visible in the 〈*u*^2^〉 as a function of temperature. The first inflection at around 100 K is caused by methyl group rotations which enter the resolution of the spectrometer [43,44]. The second inflection starting at around 200 K is related to the so-called dynamical transition. The dynamical transition of proteins was attributed to the onset of anharmonicity at 200–240 K, which is related to the melting of glassy water molecules in the hydration shell [41,45]. It was pointed out that the dynamical transition can also be explained by a finite-resolution effect of the neutron spectrometer, when the observable protein motions enter the time window of the instrument [46–48]. In our work, we calculated effective force constants 〈*k′*〉 from the linear slopes of the 〈*u*^2^〉 versus temperature in the three linear temperature ranges (table 2). Within the error bars, the 〈*u*^2^〉 at all temperatures and the force constants of human and chicken Hb as hydrated powder samples are identical. The conclusion can only be that one hydration layer is not enough to activate all motions of Hb in the *nanosecond* time scale which might be relevant for biological function. The observed difference in the dynamics between human and chicken Hb above 270 K is only activated at a hydration level larger than one hydration layer.

### 3.4. Coarse-grained Brownian dynamics simulations

To obtain a detailed understanding of the experimental observations on a residue level, supporting coarse-grained Brownian dynamics simulations of chicken and human Hb were performed. The structures of platypus and crocodile Hb are not yet available.

**Figure 6.**
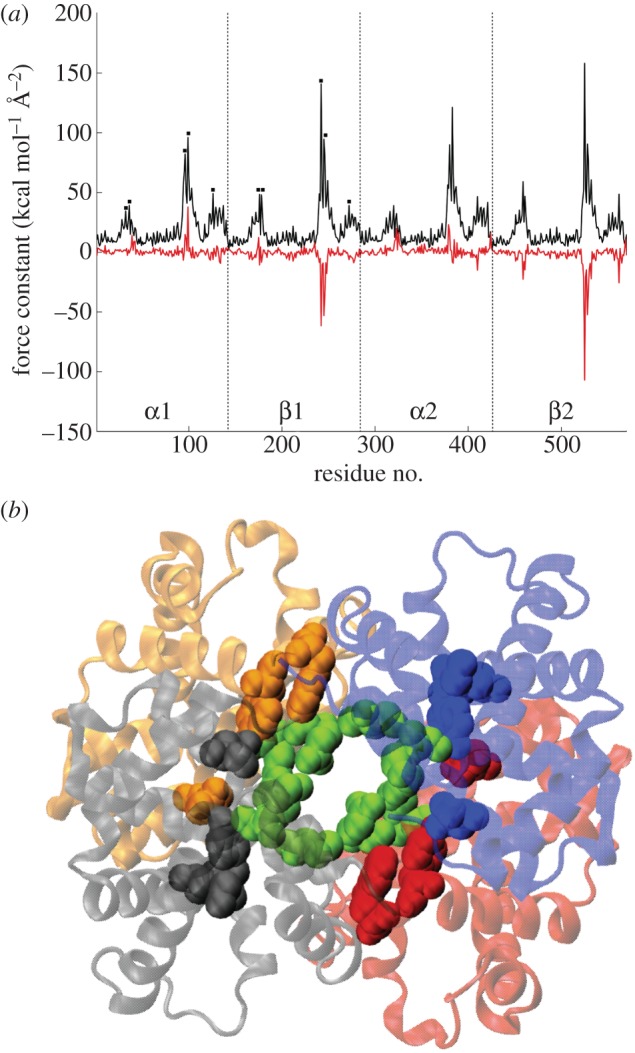
(*a*) Force-constant profile for the tetrameric biological unit of chicken Hb (black line), the black squares denote the main rigidity peaks in the first subunits and correspond to the following residues: Met32-B13, Tyr36-B17, Val96-G3, Lys99-G6 and Asp126-H9 for α1; Tyr35-B17, Trp37-C2, Glu101-G3, Arg104-G6 and Gln131-H9 for β1. Force-constant variation *Δk* = *k*_human Hb_ − *k*_chicken Hb_ of the biological unit (red line). Note that 1 kcal mol^−1^ Å^−2^ = 0.7 N m^−1^. (*b*) Cartoon representation of the biological unit of chicken Hb, with the α1, β1, α2 and β2 units plotted in red, blue, grey and orange, respectively. Green van der Waals envelopes correspond to the central rigid core (formed by G3 and G6 residues from all four units) and the remaining envelopes denote the position of rigid residues at the α1/β1 and α2/β2 interfaces.

The rigidity profile for the complete tetrameric unit of chicken deoxy Hb is represented in figure 6*a* (black line). As could be expected for this multi-domain system, the periodicity of the profile reflects the fourfold symmetry in the protein's structure and the profile is dominated by rigid residues located at the interdomain regions [13,49]. The main rigidity peaks correspond to residues Val96-G3, Lys99-G6 from the α1/2 units and residues Glu101-G3 and Arg104-G6 from the β1/2 units, which form a tight interacting network in the core of the biological unit (figure 6*b*, residues in green). Secondary rigid areas correspond to residues located around Met32-B13, Tyr36-B17 and Asp126-H9 in the α1/2 units and Tyr35-B17, Trp37-C2 and Gln131-H9 from the β1/2 units, which interact at the α1/β1 and α2/β2 interfaces (figure 6*b*, residues in red, blue, grey and orange). The variation of the force-constant profile when moving from chicken to human deoxy Hb (red line in figure 6*a*) is mainly due to a strong decrease (over −50 kcal mol^–1^ Å^–2^ = 35 N m^−1^) in the rigidity of residues Glu101-G3 and Arg104-G6 from the β1/2 units, thus suggesting that human Hb is more flexible than its chicken counterpart, notwithstanding the fact that human Hb presents a slightly denser structure than chicken Hb, reflected in the reduced models constructed for both systems, which are made of 19 930 and 19 402 springs for human and chicken Hb, respectively. We can also note that all these residues are conserved in the chicken and human Hb sequences, with the exception of Tyr36-B17 from the α-units of chicken Hb, which is replaced by a Phe residue in the human sequence. Since Tyr and Phe sidechains have similar sizes, this mutation cannot, however, be detected in our coarse-grain model for proteins.

**Figure 7.**
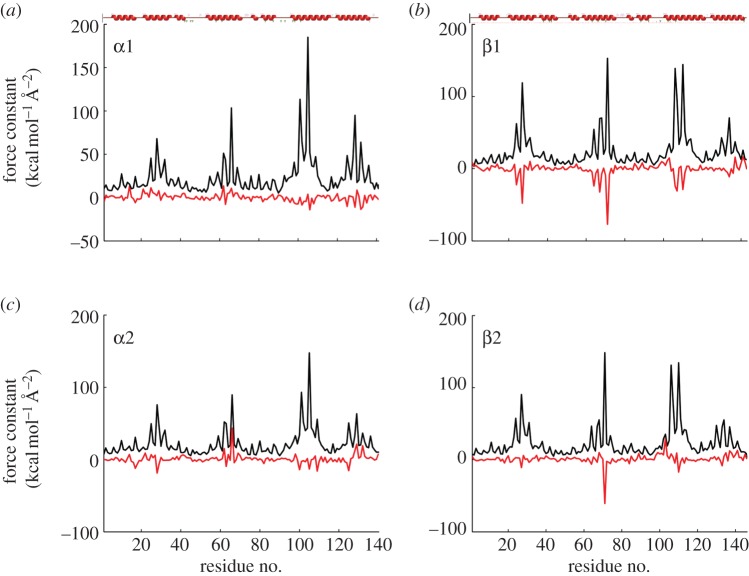
(*a*–*d*) Force-constant profiles for the four monomeric units of chicken Hb (black lines) and force-constant variations *Δk* = *k*_human Hb_ − *k*_chicken Hb_ for the monomers (red lines). The red secondary structures plot at the top of (*a*,*b*) indicate the localization of α-helices along the sequences of the α- and β-units (1 kcal mol^−1^ Å^−2^ = 0.7 N m^−1^).

Using the domain separation procedure detailed in Sacquin-Mora & Lavery [13] and Sacquin-Mora *et al*. [27], it is also possible to obtain a rigidity profile for each of the four monomers composing the tetrameric biological unit. The resulting profiles are shown in figure 7 and are qualitatively similar to those obtained in the generic study on globins performed by Bocahut *et al*. [12]. The analogous aspect of the profiles for the α- and β-subunits reflects the typical α-helical globin fold, with α-helices appearing as grouped rigidity peaks along the protein sequences and flexible regions in-between denoting, in particular, the CD and EF loops. With the exception of Leu66-E15 in the α2 subunit, all the noticeable mechanical variations that can be observed when moving from chicken to human Hb correspond to residues from the β-subunits becoming more flexible, such as Gly24-B6, Ala27-B9, Gly64-E8, Val67-E11, Phe71-E15, Leu106-G8, Gly107-G9 and Leu110-G12. Interestingly, all the aforementioned residues (which are conserved in the chicken and human Hb sequences) were previously identified as ‘Mechanically Sensitive’ residues that are located at the frontiers between internal cavities of the globin fold [12]. In particular, all four members of the globin mechanical nucleus (corresponding to position E11, E15, G8 and G12) present an important decrease of their rigidity in the β1 unit of human Hb compared with chicken Hb.

Altogether, the mechanical variations when moving from chicken to human Hb are mainly located at the central interface of the biological unit (figure 6*b*), on mechanically sensitive residues from the monomers, and only concern residues that are conserved in both sequences. Therefore, the thermal adaption of Hb internal dynamics might result from a long-range mutational effect, similar to what was previously observed in the bacterial photosynthetic reaction centre [14], where point mutations would affect the mechanical properties of residues located more than 15 Å away.
